# Arrhythmic effects of Epac‐mediated ryanodine receptor activation in Langendorff‐perfused murine hearts are associated with reduced conduction velocity

**DOI:** 10.1111/1440-1681.12751

**Published:** 2017-05-19

**Authors:** Mengye Li, Sandeep S Hothi, Samantha C Salvage, Kamalan Jeevaratnam, Andrew A Grace, Christopher L‐H Huang

**Affiliations:** ^1^ Physiological LaboratoryUniversity of CambridgeCambridgeUnited Kingdom; ^2^ Department of BiochemistryUniversity of CambridgeCambridgeUnited Kingdom; ^3^ Faculty of Health and Medical SciencesVSM BuildingUniversity of SurreyGuildfordUnited Kingdom

**Keywords:** Ca^2+^ homeostasis, cardiac arrhythmias, conduction velocity, Epac, ryanodine receptors

## Abstract

Recent papers have attributed arrhythmic substrate in murine *RyR2‐P2328S* hearts to reduced action potential (AP) conduction velocities (CV), reflecting acute functional inhibition and/or reduced expression of sodium channels. We explored for acute effects of direct exchange protein directly activated by cAMP (Epac)‐mediated ryanodine receptor‐2 (RyR2) activation on arrhythmic substrate and CV. Monophasic action potential (MAP) recordings demonstrated that initial steady (8 Hz) extrinsic pacing elicited ventricular tachycardia (VT) in 0 of 18 Langendorff‐perfused wild‐type mouse ventricles before pharmacological intervention. The Epac activator 8‐CPT (8‐(4‐chlorophenylthio)‐2′‐*O*‐methyladenosine‐3′,5′‐cyclic monophosphate) (VT in 1 of 7 hearts), and the RyR2 blocker dantrolene, either alone (0 of 11) or with 8‐CPT (0 of 9) did not then increase VT incidence (*P*>.05). Both progressively increased pacing rates and programmed extrasystolic (S2) stimuli similarly produced no VT in untreated hearts (n=20 and n=9 respectively). 8‐CPT challenge then increased VT incidences (5 of 7 and 4 of 8 hearts respectively; *P*<.05). However, dantrolene, whether alone (0 of 10 and 1 of 13) or combined with 8‐CPT (0 of 10 and 0 of 13) did not increase VT incidence relative to those observed in untreated hearts (*P*>.05). 8‐CPT but not dantrolene, whether alone or combined with 8‐CPT, correspondingly increased AP latencies (1.14±0.04 (n=7), 1.04±0.03 (n=10), 1.09±0.05 (n=8) relative to respective control values). In contrast, AP durations, conditions for 2:1 conduction block and ventricular effective refractory periods remained unchanged throughout. We thus demonstrate for the first time that acute RyR2 activation reversibly induces VT in specific association with reduced CV.

## 1 INTRODUCTION

Abnormal Ca^2+^ homeostasis increases risks of cardiac arrhythmia.^1^, ^2^ For example, increases in cardiomyocyte intracellular [Ca^2+^] arising from increased ryanodine receptor‐2 (RyR2)‐mediated sarcoplasmic reticular Ca^2+^ release activity^2^ could increase sodium–calcium exchanger activity, thereby producing delayed afterdepolarizations and premature ventricular beats.^3^, ^4^, ^5^ When superimposed upon an arrhythmic substrate, typically resulting from altered action potential (AP) conduction or recovery properties, this could trigger cardiac arrhythmias.^6^ Recent studies have reported reduced atrial^7^ and ventricular conduction velocities (CV)^8^, ^9^ that could potentially cause re‐entrant arrhythmic substrate in murine models of catecholaminergic polymorphic ventricular tachycardia (CPVT) carrying the *RyR2‐P2328S* mutation. This was attributed to acute effects of altered intracellular Ca^2+^ on Na^+^ channel (Na_v_1.5) function^7^, ^8^, ^9^ and/or sustained downregulation of Na_v_1.5 membrane expression.^9^, ^10^, ^11^, ^12^, ^13^, ^14^, ^15^, ^16^, ^17^, ^18^

The present experiments assessed whether acute manipulations of Ca^2+^ homeostasis could influence CV and introduce arrhythmic substrate in wild‐type hearts expressing normal RyR2, as opposed to *RyR2‐P2328S* hearts. They complement recent studies that had modified Ca^2+^ homeostasis through exchange protein directly activated by cAMP (Epac) activation of RyR2‐Ca^2+^ release channels^19^ through a phosphokinase A (PKA)‐independent pathway.^20^, ^21^ These studies had demonstrated that Epac activation increased Ca^2+^ spark frequencies in adult rat cardiac myocytes,^22^ amplitudes of Ca^2+^‐dependent Ca^2+^ release after isoproterenol treatment,^23^ and amplitudes and frequencies of spontaneous Ca^2+^ release in mouse ventricular cardiomyocytes.^24^ They also confirmed that Epac activation acutely increased the incidence of both VT and triggered activity,^24^ in an absence of arrhythmic substrate attributable to altered AP recovery properties. AP durations (APD) and ventricular effective refractory periods (VERPs) thus remained unchanged. However, these experiments had not investigated for changes in CV.

The present experiments therefore explored for arrhythmic susceptibility in relationship to alterations in conduction latency as a measure of CV, and to VERP and APD. It did so under conditions both of Epac activation by 8‐CPT (8‐pCPT‐2′‐O‐Me‐cAMP: 8‐(4‐chlorophenylthio)‐2′‐*O*‐methyladenosine‐3′,5′‐cyclic monophosphate),^19^ and of RyR2‐antagonism by dantrolene.^25^ The latter was both applied alone, and in combination with Epac activation by 8‐CPT. Dantrolene is known to inhibit diastolic Ca^2+^ release, decrease the frequency and duration of aberrant Ca^2+^ sparks in cultures of myocytes modelling CPVT,^26^ and heart failure.^27^, ^28^ It also increases the threshold for Ca^2+^‐induced‐Ca^2+^‐release from the sarcoplasmic reticulum in myocytes from failing but not control rabbit hearts.^28^ The present study thus additionally throws light on the use of dantrolene in a potential therapeutic approach for the management of Ca^2+^‐mediated ventricular arrhythmias.

## 2 RESULTS

**Figure 1:**
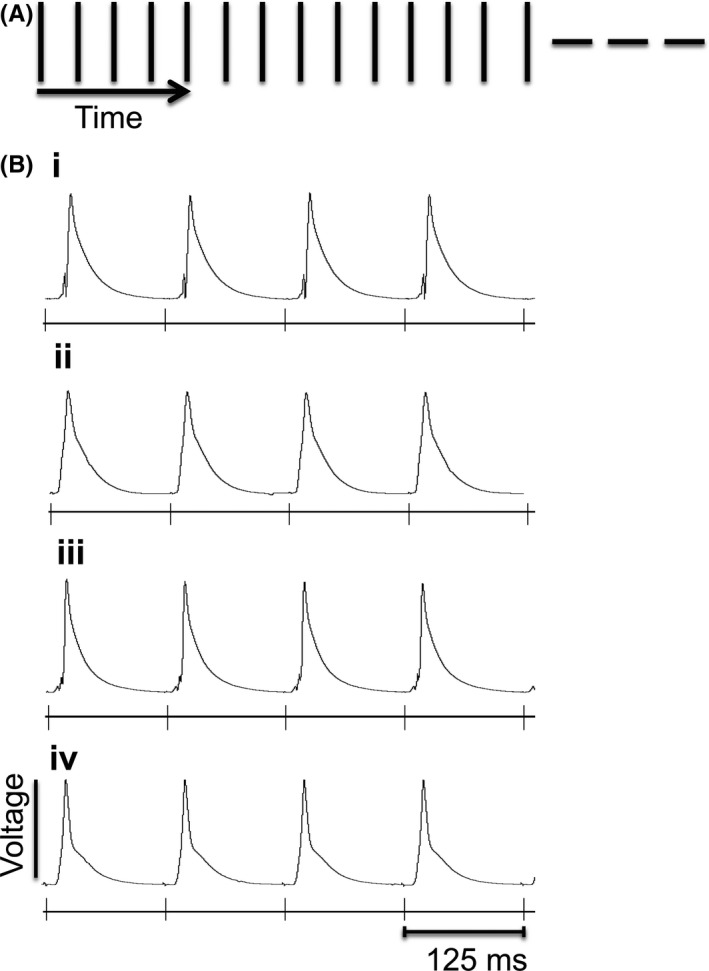
Steady (8 Hz) pacing simulating resting heart rate. (A) 8 Hz steady state protocol used to pace the heart at resting heart rate. Vertical markers below each monophasic action potential (MAP) trace marks the timing of successive pacing stimuli. (B) Typical MAP traces recorded from the left ventricle of wild‐type (WT) mice hearts perfused with either (i) control solution alone, (ii) 8‐CPT, (iii) dantrolene, or (iv) dantrolene with 8‐CPT

**Figure 2:**
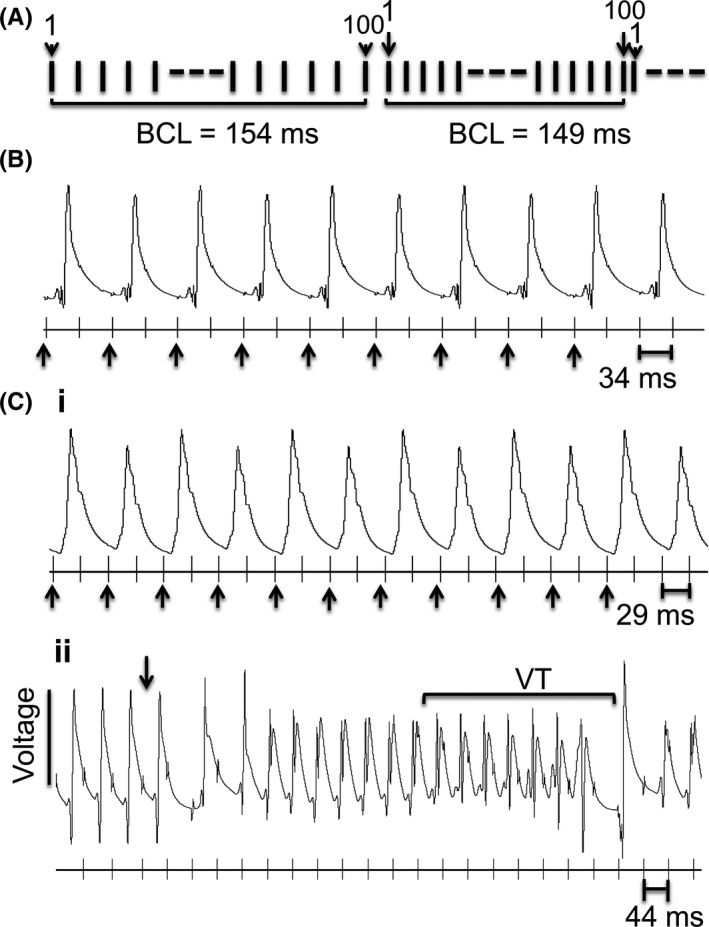
Hearts paced with the incremental pacing protocol to investigate effect of progressively increasing steady HRs. (A) An incremental pacing protocol was applied, with each stimulus train composed of 100 stimulations, starting at a basic cycle length (BCL) of 154 ms. The BCL was decreased by 5 ms for each new set of stimulus trains. Each run of the incremental pacing protocol consisted of multiple repeats of stimulus trains. The BCL decreased until it was shorter than the ventricular refractory period. This appeared as the point of onset of sustained 2:1 block (1 AP fired for every 2 stimuli), always observed in hearts perfused with control solution, dantrolene alone, or dantrolene with 8‐CPT. (B) Example of 2:1 block in a control heart. (C) In contrast, protocols performed in hearts treated with 8‐CPT may culminate in (i) 2:1 block or (ii) arrhythmia in the form of ventricular tachycardia (VT). The occurrence of each action potential is marked with an arrow below the stimulus time marking

**Figure 3:**
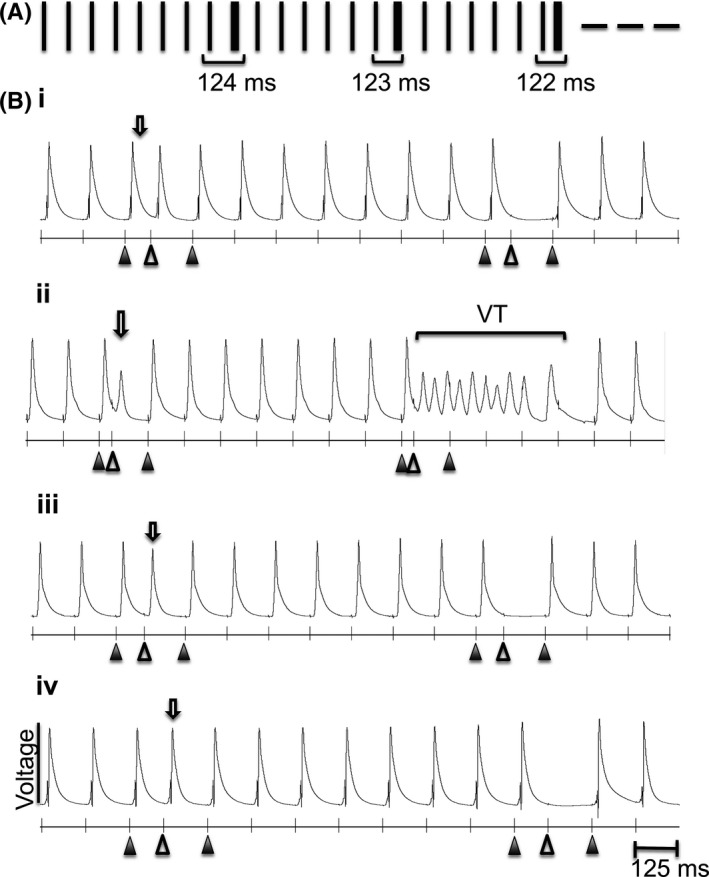
Hearts paced with the S1S2 programmed electrical stimulation (S1S2 PES) protocol to simulate the occurrence of extrasystolic beats within regular pacing. (A) The S1S2 PES protocol was composed of repeats of stimulus trains. Each stimulus is marked out by a vertical marker. Each train consisted of eight S1 stimuli ((A) narrow marker or (B) filled triangles indicating the first and last S1 stimulus in each train; BCL=125 ms), followed by an extrasystolic (S2) stimulus (wide marker in (A) and open triangle in (B)). The interval between the S2 and the preceding S1 stimulus (S1S2 interval) was shortened by 1 ms with each repetition of the S1 stimulus train. The longest S1S2 interval was 124 ms. The S1S2 PES protocol was used to study the effects of progressively shortening S1S2 stimulus intervals on the generation of arrhythmia in hearts perfused with and without drugs. Typical results compared for hearts perfused with (i) control solution, (ii) 8‐CPT, (iii) dantrolene, and (iv) with dantrolene combined with 8‐CPT treatment, showing either extrasysolic action potentials (clear arrows), refractoriness, or an episode of VT. 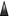: S1 stimuli; 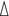: S2 stimuli; 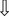: MAP in response to S2 stimulus

Figures 1, 2, 3 illustrate results from Langendorff‐perfused hearts subjected to up to three separate pacing protocols simulating different *in vivo* patterns of electrical activity in the heart. Recordings during the initial regular 8 Hz pacing stimuli, simulating *in vivo* steady state resting heart rates, corresponding to a basic cycle length (BCL) of 125 ms (Figure 1A), yielded left ventricular monophasic action potential (MAP) waveforms. Quantifications were performed only on MAP traces that fulfilled three criteria: (i) a clear established baseline preceding the MAPs; (ii) any stimulus artifact that was present being transient, with a rapid upstroke and falling phase; and (iii) repolarization from the peak of the MAP back to the baseline being monophasic. The vertical markers below each MAP trace (Figure 1B) indicate timings of successive pacing stimuli.

Results were first compared between hearts studied under control conditions (Figure 1B(i)), and following exposure to Epac activation by 8‐CPT (8‐pCPT‐2′‐O‐Me‐cAMP: 8‐(4‐chlorophenylthio)‐2′‐*O*‐methyladenosine‐3′,5′‐cyclic monophosphate); Figure 1B(ii)). The experiments then assessed the actions of the ryanodine receptor‐2 (RyR2) inhibitor dantrolene (Figure 1B(iii)) and a combination of 8‐CPT and dantrolene (Figure 1B(iv)), as detailed in the Methods. MAP recordings were used to obtain latencies, a surrogate measure for conduction velocity (CV), action potential durations at 90% recovery (APD_90_), and permitted quantifications of the frequency of spontaneous VT. No arrhythmia was observed with this initial pacing in control experiments (0 of 18). Challenge by 8‐CPT, dantrolene or dantrolene in combination with 8‐CPT all did not increase the incidence of arrhythmia (VT in 1 of 7, 0 of 11, and 0 of 9 hearts respectively; all *P*>.05).

Figure 2A illustrates the incremental pacing protocol that consisted of multiple repeats of stimulus trains each containing 100 beats at a defined BCL. It began by imposing a BCL of 154 ms. BCL was then decreased by 5 ms for each new set of stimulus trains (Figure 2A). This simulated the effect of progressively increasing heart rates. The incremental pacing protocols culminated in either a 2:1 loss of capture (where one AP is triggered for every two consecutive stimuli: Figure 2B and C(i)), which provided a measure of refractory period, or VT (Figure 2C(ii)). In the control hearts, and hearts perfused with either dantrolene alone, or dantrolene with 8‐CPT the incremental pacing protocol always culminated in a 2:1 block at high BCLs. This is exemplified in a trace (Figure 2B) obtained from a control heart, where there are exactly 0.5 times the number of recorded MAPs compared to the number of stimuli. In contrast, incremental pacing in hearts perfused with 8‐CPT (Figure 2C) culminated in either 2:1 block (Figure 2C(i)), or in VT (Figure 2C(ii)). Waveforms of consecutive action potentials observed under such conditions of 2:1 block often showed an alternans pattern. The latter is known to presage an onset of major cardiac arrhythmias in both murine and human hearts.^29^ Figure 2C(ii) illustrates a genesis of VT: regular MAPs were recorded at a stimulus BCL of 44 ms. At the arrow, stimulus interval was reduced to 39 ms, and this was followed by an episode of irregular AP firing that eventually developed into VT (marked by square bracket).

Table 1 correspondingly demonstrates significant (*P*<.05) increases in the incidence of arrhythmia with challenge by 8‐CPT. Although dantrolene administered alone had no significant impact on incidence of VT, when applied with 8‐CPT, dantrolene reversed the arrhythmic effects of 8‐CPT (8‐CPT vs dantrolene+8‐CPT; *P*<.05; control vs dantrolene+8‐CPT; *P*>.05) (Table 1).

**Table 1:** Incidence of ventricular tachycardia (VT) observed with S1S2 PES and incremental pacing protocols

	Control	8‐CPT	Dantrolene	Dantrolene with 8‐CPT
No. of hearts showing incremental pacing induced VT	0^a^ (n=9)	5^a^, ^b^ (n=7)	0 (n=10)	0^b^ (n=10)
No. of hearts showing S2‐induced VT	0^a^ (n=20)	4^a^, ^b^ (n=8)	1 (n=13)	0^b^ (n=13)

a Indicates where there is a significant increase in occurrence of VT in the presence of 8‐CPT in comparison to control solution alone (*P*<.05).

b Indicates where there is a significant reduction in the occurrence of VT in the presence of dantrolene compared to 8‐CPT alone (*P*<.05).

Figure 3 illustrates results obtained from programmed applications of extrasystolic (S2) stimuli using the S1S2 programmed electrical stimulation (S1S2 PES) protocol, simulating effects of extrasystolic events that might provoke arrhythmia in the presence of re‐entrant substrate. This consisted of runs of repeated stimulus trains, each composed of eight stimuli paced at 8 Hz followed by an S2 stimulus. The stimulus interval between the S2 and preceding S1 stimulus (known as the ‘S1S2 stimulus interval’) was shortened by 1 ms each time the stimulus train repeated, beginning at 124 ms (Figure 3A). With shortened S1S2 intervals, hearts perfused with control solution containing no drugs (Figure 3B(i)), dantrolene (Figure 3B(iii)), and dantrolene combined with 8‐CPT (Figure 3B(iv)) became refractory to the S2 stimulation, as reflected in the absence of an MAP after the S2 stimulus. This S1S2 stimulus interval therefore corresponds to the VERP.

In contrast, with 8‐CPT treatment (Figure 3B(ii)), instead of a loss of AP firing in response to the S2 stimulus, shortening of the S1S2 stimulus interval frequently resulted in VT. During quantification of such results, the S1S2 PES protocol was always repeated at least once in the same heart under the same condition to confirm observations made during the first run of the protocol. In agreement with results from the incremental pacing protocol, the S1S2 PES procedure demonstrated that challenge by 8‐CPT (*P*<.05) but not by dantrolene alone (*P*>.05) significantly increased arrhythmic incidence but that dantrolene reversed these arrhythmic effects of 8‐CPT (8‐CPT vs dantrolene+8‐CPT, *P*<.05; control vs dantrolene+8‐CPT, *P*>.05) (Table 1).

Determinations of control AP parameters prior to 8‐CPT challenge gave control values of latency (16.81±1.20 ms, n=7), APD_90_ (43.99±4.42 ms, n=5), VERPs (43.00±6.63 ms, n=7), and BCL values at the onset of 2:1 block during incremental pacing (41.00±4.54 ms (n=4)). Values of APD_90_ and VERPs had also been measured by the earlier study^24^ and agree with the present findings. Results from the subsequent experimental manouevres were accordingly normalized to control values.

Table 2 explores for changes in AP parameters as a result of challenge using 8‐CPT, dantrolene, or dantrolene in combination with 8‐CPT, by normalizing each test value against the corresponding control value in the same heart before pharmacological challenge. The resulting values of latency, APD_90_, BCL at onset of 2:1 block, and VERP were statistically assessed for significant differences. It was seen that 8‐CPT significantly increased AP conduction latency (vs controls: *P*<.01; n=7). However, it had no effect on the AP recovery parameters of APD_90_, BCL at onset of 2:1 block, and VERP (vs control: all *P*>.05; n=5, n=4, and n=7 respectively). In contrast, dantrolene alone did not affect either the AP activation or recovery parameters (*P*>.05; latency: n=10, APD_90_: n=8; BCL at onset of 2:1 block: n=5; VERP: n=7). However, results obtained following addition of 8‐CPT combined with dantrolene demonstrated that dantrolene reversed the effect of 8‐CPT on latency. It then yielded latencies indistinguishable from the control values (dantrolene+8‐CPT *vs* control: *P*>.05; n=7) and preserved normal AP recovery parameters (dantrolene+8‐CPT *vs* control: *P*>.05; n=7).

**Table 2:** Action potential (AP) parameters following pharmacological treatment normalised to control values in the same heart

Activation parameter	8‐CPT	Dantrolene	Dantrolene + 8‐CPT
Latency	1.14±0.04 (n=7)^a^	1.04±0.03 (n=10)	1.09±0.05 (n=8)
Recovery parameter			
APD_90_	1.09±0.10 (n=5)	1.11±0.07 (n=8)	1.23±0.12 (n=6)
BCL at onset of 2:1 block	0.92±0.05 (n=4)	0.96±0.02 (n=5)	0.93±0.08 (n=5)
VERP	1.10±0.11 (n=7)	0.99±0.13 (n=7)	0.95±0.13 (n=6)

APD, action potential duration. BCL, basic cycle length; VERP, ventricular effective refractory period.

a Indicates where there is a significant increase in the ratio of AP conduction latency following treatment with 8‐CPT compared prior to treatment in the control condition, as shown by an increase in ratio of latency post‐treatment vs pre‐treatment (*P*<.01; n=7).

Together these findings suggest actions of Epac‐mediated RyR2 activation by 8‐CPT both increases the incidence of arrhythmia following extrasystolic, and incremental pacing and reduces CV without affecting AP recovery properties. The RyR2 blocker dantrolene did not produce such changes. However, it reversed both the pro‐arrhythmic actions of 8‐CPT, and its actions on CV. The findings are thus consistent with acute pro‐arrhythmic actions of RyR2 receptor modulation on both CV and arrhythmia, adding to previous studies implicating chronic alterations in Na^+^ channel protein expression reported with genetic modifications of RyR2.

## 3 DISCUSSION

This study explored the effects of acute interventions involving the ryanodine receptor (RyR2)‐Ca^2+^ release channel on arrhythmogenesis and its relationship to alterations in AP conduction velocity (CV) in isolated perfused wild‐type (WT) murine hearts. Previous studies had reported that: (i) The *RyR2‐P2328S* modification, associated with human catecholaminergic polymorphic ventricular tachycardia (CPVT) resulted in increased arrhythmic tendency and increased RyR2‐mediated Ca^2+^ release in murine ventricles.^30^ (ii) This was accompanied by reductions in the CVs of both ventricular^7^ and atrial APs that could potentially create arrhythmic substrates.^8^, ^9^ (iii) The altered Ca^2+^ homeostasis also appeared to modify the Na^+^ current with consequences for CV. The latter could involve (a) a long term downregulation of cardiac Na^+^ channel (Na_v_1.5) expression that was observed in both atrial^9^ and ventricular *RyR2‐P228S* myocytes.^10^ WT rat cardiomyoctes similarly showed increased and decreased surface membrane expression of functionally active Na_v_1.5, Na_v_1.5 mRNA and total Na_v_1.5 protein following challenge by the Ca^2+^ channel blocker verapamil and the Ca^2+^ ionophore calcimycin respectively.^11^, ^12^ (b) Alternatively, acute effects of altered intracellular Ca^2+^ resulting from enhanced RyR2‐mediated Ca^2+^ release might modify Na^+^ channel function. Nav1.5 thus contains two potentially directly Ca^2+^‐sensitive regions: (i) a Ca^2+^‐binding EF‐hand motif^13^ and (ii) a calmodulin‐binding IQ domain in the Na_v_1.5 C‐terminal region.^14^, ^15^ Both these sites have been suggested to mediate Ca^2+^‐mediated modulation of Na^+^ channel function. Thus, acute increases and decreases in Na^+^ current densities followed increases or decreases in intracellular [Ca^2+^]. This was demonstrated in patch‐clamped cultured neonatal rat myocytes using buffering by BAPTA (1,2‐bis(o‐aminophenoxy)ethane‐N,N,N′,N′‐tetraacetic acid tetrakis‐acetoxymethyl ester) and caffeine administration respectively.^16^, ^17^ (iii) Additionally, Ca^2+^ may indirectly affect Na_v_1.5 through the action of calmodulin kinase II (CamKII) on multiple phosphorylation sites on the channel.^18^

The present experiments explored the effects of acute RyR2‐Ca^2+^ channel activation through the phosphokinase A (PKA)‐independent Epac^20^, ^21^ in producing VT. The experiments employed 8‐CPT ((8‐pCPT‐2′‐O‐Me‐cAMP: 8‐(4‐chlorophenylthio)‐2′‐*O*‐methyladenosine‐3′,5′‐cyclic monophosphate) as Epac agonist. Despite being a cAMP (3′5‐cyclic adenosine monophosphate) analogue, the 2‐hydroxyl group substitution on its cAMP ribose moiety disrupts its interaction with PKA whilst permitting binding to Epac. This gives 8‐CPT a 300‐fold selectivity for Epac relative to PKA at the low, 1 μmol/L, concentrations employed here.^19^, ^31^, ^32^, ^33^ Further reported actions of 8‐CPT on phosphodiesterase isoforms required significantly higher inhibitory binding concentrations than those used here.^34^

Epac is implicated in adrenergically‐mediated cardiac arrhythmogenesis.^22^ Previous studies reported that experimental Epac activation increased the frequencies and amplitudes of spontaneous Ca^2+^ release^22^, ^24^ and the amplitudes of Ca^2+^ waves resulting from Ca^2+^‐induced Ca^2+^ release.^23^ It was arrhythmogenic in Langendorff‐perfused whole hearts,^24^ likely through altered Ca^2+^ homestasis following its interaction with and opening of the RyR2‐Ca^2+^‐release channel.^19^ The present experiments nevertheless first confirmed that 8‐CPT acutely produced arrhythmic substrate. Admittedly, steady extrinsic pacing, whether before or following pharmacological intervention, did not induce arrhythmia, but nevertheless provided the AP parameters of APD and latency. Nevertheless, both extrinsic pacing at progressively increased frequencies and programmed applications of extrasystolic (S2) stimuli induced VT in 8‐CPT‐treated but not untreated hearts during monophasic AP recordings. These also provided indications as to whether alterations in refractoriness properties took place. The protocols therefore together permitted the presence of absence of arrhythmia to be correlated with the corresponding presence or absence of alterations in particular AP parameters.

Secondly, the arrhythmic effects of 8‐CPT challenge were accompanied by a slowed CV as reflected in changes in AP latencies. However, it did not affect the AP recovery properties of APD and VERP.

Thirdly, pharmacological manipulations using the RyR2 antagonist dantrolene corroborated these findings. Multiple studies have demonstrated that dantrolene reduces RyR2‐mediated Ca^2+^ leak, decreasing the incidence and duration of Ca^2+^ sparks in cultures of cardiomyocytes modelling both CPVT^26^ and cardiac failure.^27^, ^28^ Dantrolene may enhance the interactions between the N‐terminal and the central domain of the RyR2 molecule. These interactions stabilise the closed state of the channel.^26^ Furthermore dantrolene shows an increased binding following modification of the RyR2 conformation by both EGTA, and during cardiac failure. Thus, dantrolene inhibited RyR2 Ca^2+^ release particularly under conditions of increased channel open probability.^26^, ^27^, ^35^ The present experiments demonstrated that applied by itself, dantrolene altered neither arrhythmic tendency nor AP conduction and recovery parameters. However, it reversed the actions of 8‐CPT. Thus when both drugs were added in combination, there was no arrhythmia, and AP parameters were restored to normal.

Together these findings suggest that reversible arrhythmic effects related to slowed AP propagation but unchanged AP recovery can follow acute modifications of Ca^2+^ homeostasis accompanying interventions increasing RyR2‐mediated sarcoplasmic reticulum Ca^2+^ release. They complement previous observations of a longer term reduction in Nav1.5 expression following chronic alterations in Ca^2+^ homeostasis associated with the *RyR2‐P2328S* mutation.^10^

## 4 METHODS

All animals used for experiments were of the WT sv129 background. A total of 20 male and female, between the ages of 5 and 7 months, were used for the experiments. Mice were kept in animal house facilities at 21°C and exposed to 12 hour dark : light cycle each day, with free access to sterile rodent chow, drinking water, and bedding at all times. All animals were killed by cervical dislocation by Home Office‐licensed personnel. Procedures used conformed to Schedule 1 of the UK Animals (Scientific Procedures) Act (1986), and to Institutional Ethics Committee Guidelines. Procuration of animals, their husbandry and all experiments also conformed to the ‘European Convention for the Protection of Vertebrate Animals used for Experimental and other Scientific Purposes’ (Council of Europe No 123, Strasbourg 1985). The hearts were isolated from the animal by bilateral sternotomy and placed upon ice‐cold Krebs‐Henseleit (KH) solution (pH 7.4; NaCl [119 mmol/L], NaHCO_3_ [25 mmol/L], KCl [4 mmol/L], MgCl_2_ [1 mmol/L], KH_2_PO_4_ [1.2 mmol/L], CaCl_2_ [1.8 mmol/L], glucose [10 mmol/L] and sodium pyruvate [1.8 mmol/L]); the solution was bubbled through with 95% O_2_ and 5% CO_2_ (British Oxygen Company, Manchester, UK).

The heart was dissected free of connective tissue to expose 3‐4 mm of aorta. The aorta was cannulated with a tailor‐made 21‐gauge cannula pre‐filled with cold KH solution, and a micro‐aneurysm clip (Harvard Apparatus, Cambridge, UK) was used to fix the cannula into place. Thereafter, the heart was quickly placed within the perfusion system. KH solution used to perfuse the heart was first passed through a 200 μm followed by a 5 μm filter, and warmed to 37°C by a water jacket and circulator. A peristaltic pump (Watson‐Marlow Bredel pumps model 505S, Falmouth, Cornwall, UK) was used to deliver the perfusion fluid at a constant rate of 2.3 mL/min. After commencement of continuous perfusion, hearts were investigated for spontaneous contractions and confirmatory signs of viability.

Perfused hearts were extrinsically paced using a bipolar Ag/AgCl stimulating electrode placed on the right ventricular epicardium. The stimulus voltage on the constant voltage stimulator (stimulator model DS2A‐MkII; Digitimer, Welwyn Garden City, Herts, UK) was set to deliver stimuli at double the threshold voltage. The isolated Langendorff‐perfused WT murine hearts were paced at 8 Hz for at least 15 minutes before beginning MAP recordings from the left ventricular epicardium, midway between ventricular apex and base, using a MAP electrode (Linton Instruments, Linton, Cambs, UK). The relative dimensions of the MAP electrodes and of the murine ventricles permitted only a single channel of MAP recording at any given time The recorded data was amplified with a NL100AK head stage pre‐amplifier, followed by a NL104A amplifier (Digitimer). The signal was then band pass filtered (filter range 0.5 Hz‐1 kHz; Neurolog model NL125/6 filter; Digitimer) and digitised (1401plus MKII; Cambridge Electronic Design, Cambridge, UK). All MAP traces were recorded electronically, and the waveforms analyzed using Spike2 software (Cambridge Electronic Design).

Experiments were carried out under the following conditions, defined by the perfusate used for the heart.

1. The control procedure used KH solution alone without any added pharmacological agents.
2. 8‐CPT (8‐pCPT‐2′‐O‐Me‐cAMP: 8‐(4‐chlorophenylthio)‐2′‐*O*‐methyladenosine‐3′,5′‐cyclic monophosphate; BIOLOG Life Science Institute, Bremen, Germany) was made into a stock of 1 mmol/L with dimethyl sulfoxide (DMSO; stored at −20°C). When needed for use, the stock was then diluted in KH solution to make up a final concentration of 1 μmol/L 8‐CPT, in accord with that previously used in the earlier study.^24^
3. Dantrolene sodium (Sigma‐Aldrich, Poole, Dorset, UK) was dissolved in DMSO to make a 5 mmol/L stock (stored at 4°C). The final concentration of 5 μmol/L of dantrolene used in experiments were obtained by dilution of the stock with KH solution. Following control recordings, the pharmacological manoeuvres involved applications of either 8‐CPT alone (1 μmol/L) followed by treatment with both 8‐CPT and dantrolene (1 μmol/L and 5 μmol/L respectively), or dantrolene alone (5 μmol/L) followed by treatment with both drugs. During these procedures, hearts were paced at 8 Hz for at least 3 minutes after the estimated time of drug arrival at the heart before recordings were made.

All data are expressed as mean±SEM; where time is the variable, data is presented in milliseconds (ms). The number of hearts from which data is derived from for each condition is designated *n*. For categorical data, Fisher's exact test was used to test for statistical significance between two data sets, each with two possible outcomes, the presence or absence of VT. Changes in the continuous parameters of AP latency, AP duration at 90% recovery (APD_90_), basic cycle length (BCL) at the onset of 2:1 block, and VERPs were tested using Student's paired *t*‐test. Statistical significance obtained from the t‐test was defined as *P*<.05.

## DISCLOSURE

None declared.
